# Piphillin predicts metagenomic composition and dynamics from DADA2-corrected 16S rDNA sequences

**DOI:** 10.1186/s12864-019-6427-1

**Published:** 2020-01-17

**Authors:** Nicole R. Narayan, Thomas Weinmaier, Emilio J. Laserna-Mendieta, Marcus J. Claesson, Fergus Shanahan, Karim Dabbagh, Shoko Iwai, Todd Z. DeSantis

**Affiliations:** 1 Informatics Department, Second Genome Inc., South San Francisco, California, USA; 2 APC Microbiome Ireland, University College Cork, Co., Cork, Ireland; 3 School of Microbiology, University College Cork, Co., Cork, Ireland; 4 Department of Medicine, University College Cork, Co., Cork, Ireland

**Keywords:** Metagenomics, Phylogenetic analysis, Sequence alignment, Shotgun sequencing, Genomic databases

## Abstract

**Background:**

Shotgun metagenomic sequencing reveals the potential in microbial communities. However, lower-cost 16S ribosomal RNA (rRNA) gene sequencing provides taxonomic, not functional, observations. To remedy this, we previously introduced Piphillin, a software package that predicts functional metagenomic content based on the frequency of detected 16S rRNA gene sequences corresponding to genomes in regularly updated, functionally annotated genome databases. Piphillin (and similar tools) have previously been evaluated on 16S rRNA data processed by the clustering of sequences into operational taxonomic units (OTUs). New techniques such as amplicon sequence variant error correction are in increased use, but it is unknown if these techniques perform better in metagenomic content prediction pipelines, or if they should be treated the same as OTU data in respect to optimal pipeline parameters.

**Results:**

To evaluate the effect of 16S rRNA sequence analysis method (clustering sequences into OTUs vs amplicon sequence variant error correction into amplicon sequence variants (ASVs)) on the ability of Piphillin to predict functional metagenomic content, we evaluated Piphillin-predicted functional content from 16S rRNA sequence data processed through OTU clustering and error correction into ASVs compared to corresponding shotgun metagenomic data. We show a strong correlation between metagenomic data and Piphillin-predicted functional content resulting from both 16S rRNA sequence analysis methods. Differential abundance testing with Piphillin-predicted functional content exhibited a low false positive rate (< 0.05) while capturing a large fraction of the differentially abundant features resulting from corresponding metagenomic data. However, Piphillin prediction performance was optimal at different cutoff parameters depending on 16S rRNA sequence analysis method. Using data analyzed with amplicon sequence variant error correction, Piphillin outperformed comparable tools, for instance exhibiting 19% greater balanced accuracy and 54% greater precision compared to PICRUSt2.

**Conclusions:**

Our results demonstrate that raw Illumina sequences should be processed for subsequent Piphillin analysis using amplicon sequence variant error correction (with DADA2 or similar methods) and run using a 99% ID cutoff for Piphillin, while sequences generated on platforms other than Illumina should be processed via OTU clustering (e.g., UPARSE) and run using a 96% ID cutoff for Piphillin. Piphillin is publicly available for academic users (Piphillin server. http://piphillin.secondgenome.com/.)

## Background

Several recently published reports on studies of the microbiome have utilized 16S rRNA gene sequencing to investigate the taxonomic composition of bacterial communities and link the abundance of certain microbial taxa to host characteristics, such as inflammation or fluctuations in metabolism [1, 2]. However, many associations between the microbiome and symptoms of clinical disease might stem from the microbiome’s breadth of microbial metabolic functions, not from its taxonomic content. Unlike shotgun metagenomic sequencing, which surveys entire genomes within a microbial sample, 16S rRNA gene sequencing provides no data regarding the functional capacity of a microbial community. While metagenomics approaches are oftentimes ideal, they require extremely deep sequencing to adequately profile the functional content of a bacterial community [3], are prohibitively expensive, and are simply impractical for many researchers.

Predicting microbial functional content from 16S rRNA gene sequencing data is a popular alternative to shotgun metagenomic approaches. In 2016, Piphillin was introduced: a publicly available software capable of predicting microbial functional content from the presence of detected 16S rRNA genes. With Piphillin, representative nucleic acid sequences from candidate operational taxonomic units (OTU) are compared directly with 16S rRNA gene sequences from genomes in the database to infer genome content, and thus functional potential [4]. After having corrected for copy number, Piphillin uses direct nearest-neighbor matching of 16S rRNA gene amplicons with genomes from reference databases (e.g.*,* Kyoto Encyclopedia of Genes and Genomes (KEGG), BioCyc) to predict metagenomic content. Piphillin has been broadly used by more than 350 researchers worldwide to study a wide array of microbial environments: including skin [5] and soil microbiomes [6]. Piphillin has distinct advantages over competing tools: Piphillin does not rely on phylogenetic trees to infer metagenomic data and is amenable to frequent database changes (having been updated with six new databases since its inception). Both the KEGG and BioCyc databases have substantially expanded since Piphillin’s initial release, the former having gained 1346 genomes and the latter 3038 genomes (36 and 69% increases in overall database size, respectively). Since Piphillin exploits nearest-neighbor matching of 16S rRNA gene sequences to genomic sequence data held in these databases, the significant expansion observed in both collections increases the likelihood of matched candidates. Therefore, these expansions enhance the integrity and accuracy of predicted genome contents. Considering these significant changes to reference sequence databases, it is necessary to re-assess Piphillin using the same metrics and criteria described in the original paper.

In 2016, Piphillin-predicted gene abundance data had significantly higher correlation with metagenomic data compared to PICRUSt-(Phylogenetic Investigation of Communities by Reconstruction of Unobserved States) generated data. Furthermore, when compared directly to the results of PICRUSt, differential abundance analyses of Piphillin-predicted data yielded a 15% increase in balanced accuracy [4, 7]. In the original publication Piphillin also compared favorably against Tax4Fun, another package predicting functional profiles from 16S rRNA sequence data.

In 2018, the beta version of PICRUSt2 was released as a successor of PICRUSt. The original PICRUSt was bound by limitations, including the requirement for QIIME for 16S rRNA sequence analysis and dependence on reference phylogenetic trees, which hindered reference database updates [4]. Despite its newer, updated database, PICRUSt2 continues to use a reference phylogenetic tree. While PICRUSt2 overcame some of these limitations *(*i.e.*,* a substantial update to its reference database), the platform still relies heavily on phylogenetic trees (the utility of which in this context has been debated [8]). PICRUSt2 also requires a significantly larger computational investment than Piphillin: requiring a minimum of 16 GB of RAM. Currently, Piphillin is available through a web application, so users have no computational hurdle for use. Ribosomal RNA gene sequence data can be imported into both Piphillin and PICRUSt2 in multiple formats, including clustering into OTU and DADA2-corrected amplicon sequence variants (ASV).

Traditionally, 16S rRNA gene sequence analyses have been performed by first trimming reads and then clustering sequences to (i) an external reference (closed-reference OTU picking), (ii) de novo OTUs based on 97% similarity, or (iii) an external reference followed by de novo OTU clustering on remaining reads (open-reference OTU picking [9, 10]). Pipelines like DADA2 use sequence error models to correct amplicon errors into ASVs. These techniques, capable of identifying denoised sequence variants while minimizing the identification of spurious sequences, are becoming a popular approach for analyzing 16S rRNA gene sequence data [11, 12]. Like PICRUSt2, Piphillin is capable of predicting metagenomic content from DADA2-corrected ASVs. However, while ASV-based sequence error correction and similar methods have gained in popularity for analyzing 16S rRNA gene sequence data, it remains unclear how these techniques perform in metagenomic functional content prediction pipelines like Piphillin and PICRUSt2.

Here we show that Piphillin, equipped with the most up-to-date database, still shows high correlation to corresponding metagenomic data, using either 97% de novo clustered OTUs or DADA2-corrected ASVs as input data. In differential abundance testing, Piphillin from DADA2-corrected ASVs is also shown to yield 19% greater balanced accuracy than PICRUSt2. Additionally, updates of the Piphillin reference database allow for more sequences to be identified during Piphillin analysis. We also show the capacity of BioCyc as a reference to provide metagenomic predictions better correlated to shotgun metagenomics results in an environmental dataset. Finally, we introduce the new release of Piphillin, v7.0, which includes additional features such as tables showing the contribution of each individual genome to metagenomic predictions.

## Results

### 16S rRNA sequence analysis approach impacts the quantity of sequences kept for processing, correlation to metagenomic data, and detection of differentially abundant features

**Fig. 1:**
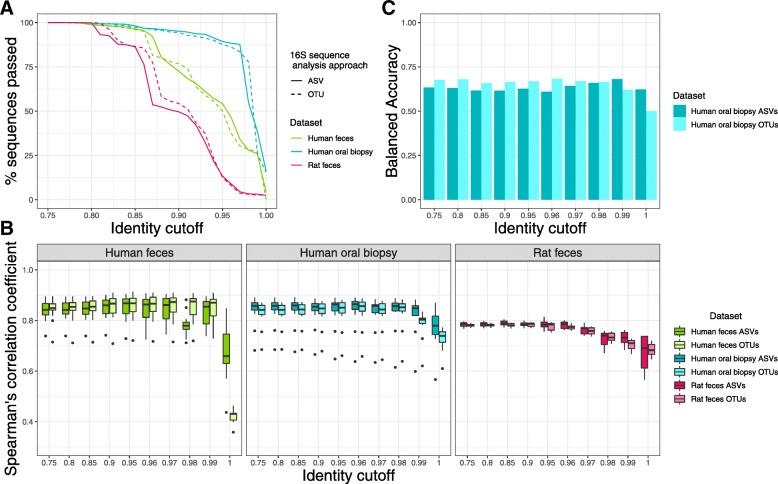
Piphillin results comparing 16S rRNA sequence analysis approaches using the KEGG database. **a** 16S rRNA gene amplicon sequences passing the identity threshold to the reference genomes. Percentage of amplicon sequences from two datasets using two different 16S rRNA sequence analysis approaches passing identity cutoffs from 75 to 100% against 16S rRNA gene sequences in the KEGG genome database. **b** Spearman’s correlation coefficient between Piphillin results and shotgun metagenomics at ten different identity cutoffs tested in Piphillin. Spearman’s correlation coefficient was calculated for each sample and mean, 1st and 3rd quartiles are depicted by the boxes. Whiskers extend to the furthest points within 150% of the interquartile range. **c** Balanced accuracy in identifying differentially abundant KOs from Piphillin against corresponding metagenomics at each identity cutoff. * indicates *p* < 0.05, ** indicates *p* < 0.001, *** indicates *p* < 0.0001

**Fig. 2:**
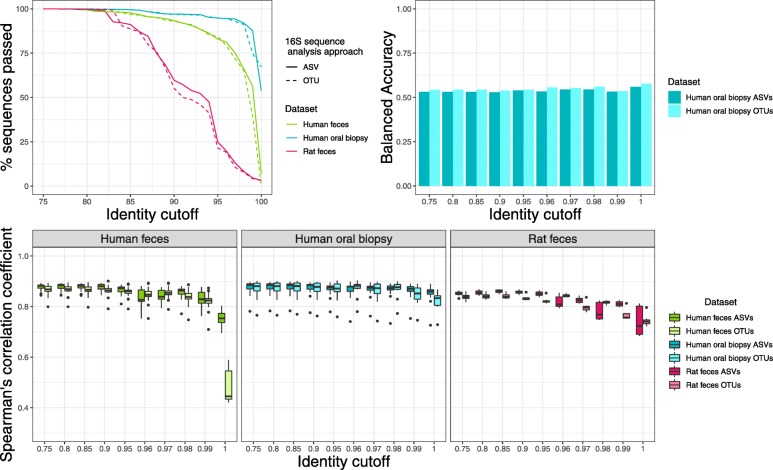
Piphillin results comparing 16S rRNA sequence analysis approaches using the BioCyc database. **a** 16S rRNA gene amplicon sequences passing the identity threshold to the reference genomes. Percentage of amplicon sequences from two datasets using two different 16S rRNA sequence analysis approaches passing identity cutoffs from 75 to 100% against 16S rRNA gene sequences in the BioCyc genome database. **b** Spearman’s correlation coefficient between Piphillin results and shotgun metagenomics at ten different identity cutoffs tested in Piphillin. Spearman’s correlation coefficient was calculated for each sample and mean, 1st and 3rd quartiles are depicted by the boxes. Whiskers extend to the furthest points within 150% of the interquartile range. **c** Balanced accuracy in identifying differentially abundant features from Piphillin against corresponding metagenomics at each identity cutoff. * indicates *p* < 0.05, ** indicates *p* < 0.001, *** indicates *p* < 0.0001

Traditionally, 16S rRNA gene sequence data has been analyzed via either clustering sequences to an external reference (closed-reference OTU picking), clustering sequences to an external reference then de novo OTU clustering on remaining reads (open-reference OTU picking), or de novo OTU clustering on all reads. Other approaches, such as DADA2, correct exact amplicon sequence variants by modeling and correcting sequence errors. We studied the impact of 16S rRNA gene sequence analysis method (ASV error correction with DADA2 (ASVs) versus 97% de novo OTU clustering using UPARSE (OTUs)) on Piphillin results at varying identity cutoffs. We predicted corresponding metagenomes using three datasets: human feces, human oral biopsy, and rat fecal samples. The hypersaline microbial mat dataset used later in this analysis was not processed with DADA2 as only fasta files *without* quality scores were available. For Piphillin predictions using the KEGG database using either ASVs or OTUs, a similar proportion of sequences matched to the KEGG database and were subsequently used for metagenomic prediction (Fig. 1a). When using the BioCyc database, OTUs and ASVs input yielded similar results with regard to sequences used in analysis (Fig. 2a).

To examine how different 16S rRNA gene sequence analysis approaches impact Piphillin metagenomic content prediction, we calculated the Spearman’s correlation coefficient between Piphillin results and corresponding shotgun metagenomics data from the three datasets. When querying the Piphillin KEGG database, outputs from ASVs and OTUs correlated to corresponding shotgun metagenomics data in similar fashion when sequence identity cutoffs ranged from 75 to 97% (Fig. 1b). However, at 100 and 99% identity cutoffs for the human oral dataset, correlations were significantly stronger from ASVs than from OTUs. For the human feces dataset, Piphillin data from ASVs had a significantly stronger correlation than Piphillin data stemming from OTUs at 100% ID cutoff, but the opposite was true at 98%. When using the BioCyc database, Piphillin outputs from ASVs and from OTUs correlated similarly in the human oral biopsy dataset (Fig. 2b). However, Piphillin outputs from ASVs for rat feces yielded significantly stronger correlation values to metagenomics results than OTUs-based Piphillin results for identity cutoffs 80, 85, 90, 95, 97, and 99% (Fig. 2b). The same was also true for the human feces at 75, 80, 85, 90, 98, and 100% (Fig. 2b).

Most microbiome studies are predicated on the attempt at identifying differentially abundant characteristics between two groups (e.g.*,* treatment versus control). We examined the impact of two different 16S rRNA gene sequence analysis strategies on the ability of Piphillin-predicted functional content to approximate differential abundance patterns in corresponding metagenomic data. Corresponding KEGG-reference aligned metagenomic and Piphillin-predicted data were used at various identity cutoffs from human oral biopsies to assess differential abundance. When comparing results from the Piphillin-predicted data to the metagenomic data, balanced accuracy of differential abundance analysis ranged from 0.61–0.68 and 0.50–0.68 for ASVs and OTUs, respectively (BA = TPR / 2 + (1-FPR) / 2, TPR = true positive rate, FPR = false positive rate). Peak balanced accuracy for Piphillin using ASVs was at 99%, and was 96% for OTUs, with a balanced accuracy of 0.68 for both (Fig. 1c). For OTUs input, the balanced accuracy at 96% identity was only 0.02 higher than the balanced accuracy at 97% identity, which was the recommended identity cutoff in our original paper. When the same analysis was conducted using BioCyc as a reference database, results were not as promising. Piphillin-predicted metagenomes yielded lower balanced accuracy (0.53–0.58), irrespective of 16S rRNA gene sequence analysis approach employed or Piphillin identity cutoff instated (Fig. 2c).

### Piphillin yields greater balanced accuracy than PICRUSt2 in differential abundance analysis

**Fig. 3:**
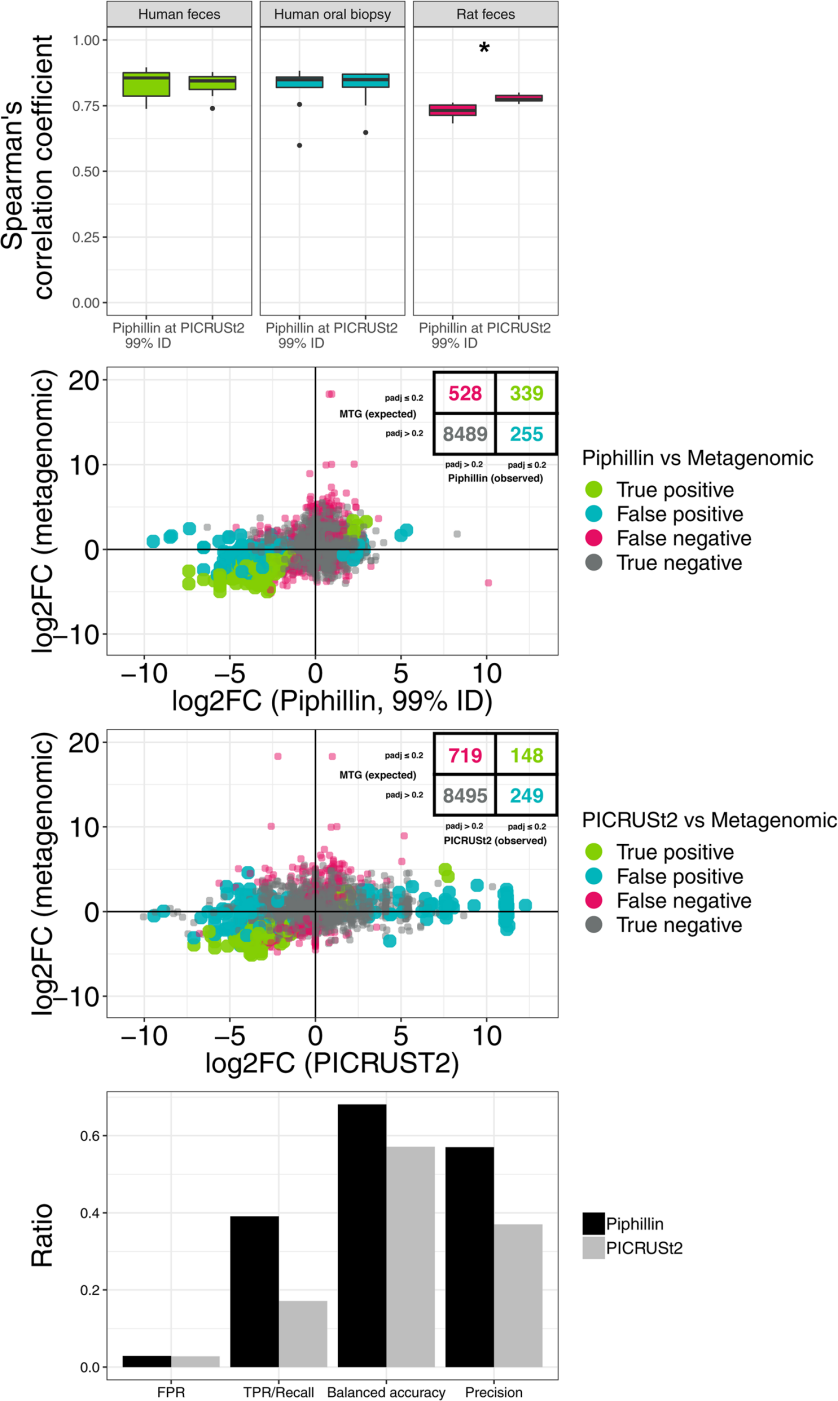
Comparison between Piphillin and PICRUSt2 using DADA2-corrected ASV data. **a** Spearman’s correlation coefficient against corresponding shotgun metagenomics results were compared for two datasets. Spearman’s correlation coefficient was calculated for each sample and ranges are depicted as box and whisker plots as described in Fig. 1. **b** Comparison of log2FC in differential abundance analysis of KOs between metagenomic and Piphillin-predicted data. Color based on comparison to metagenomics results, in which the adjusted *p*-value cutoff was 0.2 for significance. **c** Comparison of log2FC in differential abundance analysis of KOs between metagenomic and PICRUSt2-predicted data. Color based on metagenomics results, in which the adjusted p-value cutoff was 0.2 for significance. **d** False positive rate, true positive rate/recall, balanced accuracy, and precision of detecting significant differences between cancer and healthy human oral biopsy samples were compared. * indicates *p* < 0.05, ** indicates *p* < 0.001, *** indicates *p* < 0.0001

There are several bioinformatic tools available for predicting functional content from 16S rRNA gene sequence data, although many are not currently being maintained. We previously compared Piphillin to PICRUSt, which uses phylogeny to predict genomic content. Its successor, PICRUSt2, is publicly available and in beta testing as of 2018. To compare performance between Piphillin and PICRUSt2, we employed both utilities to predict functional content from DADA2-corrected ASVs from human feces, human oral biopsies, and rat feces using KEGG reference databases available for each pipeline. Since Piphillin results from ASVs yielded the greatest balanced accuracy at the 99% sequence identity cutoff, we used these results in the direct comparison. For the human feces and human oral biopsy datasets, there was no statistical difference in correlation between Piphillin- and PICRUSt2- predicted functional content and corresponding metagenomic data (Fig. 3a). However, PICRUSt2- predicted functional content correlated more strongly with corresponding metagenomic data than Piphillin (*p* < 0.05) for the rat feces dataset.

We then examined the ability to detect differential abundances between predicted genomic content and corresponding metagenomic data. Using the human oral biopsy dataset, we compared paired cancer and healthy tissue. Piphillin predicted more true positives than PICRUSt2 (339 vs 148; Fig. 3b and c). While Piphillin exhibited a slightly greater FPR (FPR = False positives / (False positives + True negatives)) than PICRUSt2, both pipelines yielded comparably low FPRs (both < 0.05; Fig. 3d). Piphillin yielded a TPR of 0.391, which was 129% greater than that of PICRUSt2 (TPR = True Positive / (True Positive + False Negative); Fig. 3d). Piphillin’s elevated TPR coincided with 19% greater balanced accuracy (BA) and 54% greater precision than PICRUSt2 (BA = TPR / 2 + (1-FPR) / 2, Precision = True positives / (True positives + False positives)); Fig. 3d).

One of the notable differences between ASVs and OTUs techniques is the denoising process, which is only available with ASVs. In addition, the ASV analysis approach can yield ASVs with strain level annotation. This method of sequence filtering by modeling oftentimes precludes a large fraction of reads from consideration in downstream analyses. After having analyzed sequences via either ASV and OTU techniques, 47 and 84% of the raw sequences were retained, respectively, for the human oral biopsy dataset. Upon executing Piphillin with ASVs at the recommended 99% cutoff, 990,327 sequences (18% of the total unprocessed reads) were able to be considered. Upon executing Piphillin with OTUs at the recommended 96% cutoff, 3,926,405 sequences (72% of the total unprocessed reads) were able to be considered. Even with the relatively low fraction of adequate initial sequences, Piphillin had similar balanced accuracy for ASV as for OTUs.

### Reference database updates increase the quantity of sequences assessed per analysis

**Fig. 4:**
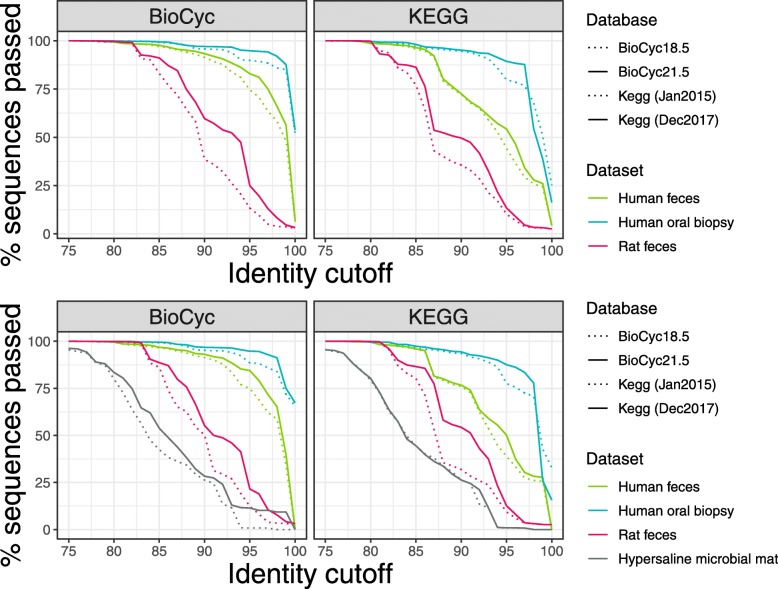
Updated references results in more sequences considered in Piphillin analysis. Comparison of 16S rRNA gene amplicon sequences passing the identity threshold to the reference genomes based on database version for Piphillin results from (**a**) DADA2-corrected ASVs and (**b**) 97% de novo OTUs

One advantage of Piphillin over other functional prediction pipelines is that the software structure allows for frequent database updates. Piphillin was initially released using the Kegg (Jan2015) and Biocyc18.5 reference databases, which contained 3036 and 4382 genomes respectively. The current Piphillin database utilizes Kegg (Dec2017) and BioCyc21.5, which contain 4132 (36% increase) and 7420 (69% increase) genomes, respectively. We hypothesized that Piphillin executed with a later version of the database would be capable of interrogating a greater number of sequences after searching nearest-neighbor genomes of candidate sequences due to the drastic increase in genome numbers in each database. Compared to the original KEGG and BioCyc reference databases available for Piphillin (Kegg (Jan2015) and Biocyc18.5), later collections provide more sequences to be compared per analysis regardless of the 16S rRNA gene sequence evaluation method performed (ASVs, Fig. 4a; OTUs, Fig. 4b). For the ASVs, datasets sequenced with the Illumina platform and analyzed with Piphillin at 99% sequence ID cutoff (the ID for peak balanced accuracy for Piphillin performed on ASVs), the updated databases resulted in the ability of up to 1.81% more sequences to be compared in the Piphillin analysis against the KEGG database, and up to 8.38% more sequences to be compared in Piphillin analysis against the BioCyc database (both from the human stool dataset; Fig. 4a).

Similar to Piphillin performed from ASVs, Piphillin performed from OTUs had more sequences considered with database updates. At 96% ID (the Piphillin sequence ID cutoff that had peak balanced accuracy when performed from OTUs), updated collections enabled up to 10.64% more sequences to be considered in Piphillin analysis against the KEGG database (in the human oral biopsy dataset), and up to 10.77% more sequences against the BioCyc database (in the rat feces dataset; Fig. 4b).

### Piphillin results generated with updated BioCyc database show significantly stronger correlation to corresponding metagenomic data in environmental samples

**Fig. 5:**
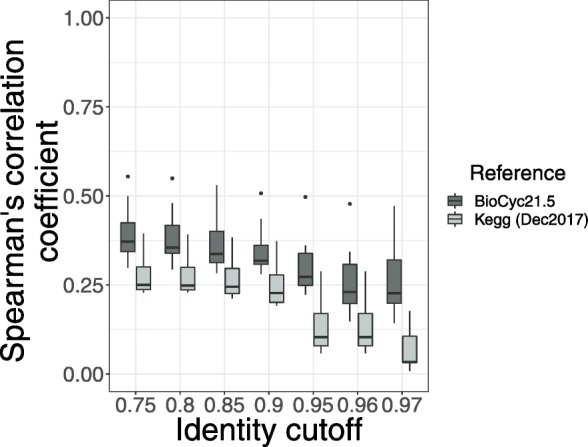
Piphillin executed with BioCyc vs KEGG reference on environmental samples. Spearman’s correlation coefficient against corresponding shotgun metagenomics results were compared the hypersaline microbial mat dataset using either KEGG and BioCyc references. Spearman’s correlation coefficient was calculated for each sample and ranges are depicted as box and whisker plots as described in Fig. 1. * indicates *p* < 0.05, ** indicates *p* < 0.001, *** indicates *p* < 0.0001

In the original manuscript, we reported that for an environmental dataset (hypersaline microbial mats), Piphillin-predicted genomic content exhibited low correlation (mean rho = 0.28 at any identity cutoff for KEGG database and 0.27 at any identity cutoff for BioCyc database) to corresponding metagenomics results. In addition, there was no significant difference between KEGG and BioCyc-derived correlation results. To evaluate improvements to Piphillin stemming from updated databases, we re-analyzed the same hypersaline microbial mat samples against both KEGG and BioCyc reference databases. Since the data available for testing did not contain base quality values, only results from OTUs were compared. Mean resulting Spearman’s correlation coefficients ranged from 0.04 to 0.28 for KEGG and 0.26 to 0.39 for BioCyc. The BioCyc reference database yielded significantly greater correlation coefficients at all identity cutoffs (75 to 97%) than its KEGG counterpart (*p* < 0.05; Fig. 5).

## Discussion

Since the original publication introducing Piphillin in 2016, the tool has been continuously updated in part through active dialogue with more than 350 users. We previously tested the ability of Piphillin to predict genomic content from de novo OTUs and found its balanced accuracy to be higher than other pipelines (e.g., PICRUSt, Tax4Fun) [7, 13]. However, little was known regarding Piphillin’s compatibility with other 16S rRNA gene sequencing strategies, such as amplicon sequence variant error correction with DADA2. Moreover, Piphillin had not been directly compared to contemporary comparable pipelines, such as PICRUSt2.

Metagenomic functional prediction derived from empirical 16S rRNA gene sequence data is a possible alternative to metagenomic shotgun sequencing. This capability, however, is not without limitations and is predicated on the assumption that bioinformatic utilities like Piphillin can adequately reconstruct metagenomic data, regardless of the method by which 16S rRNA gene sequences were generated and processed. To test this assumption, we directly compared Piphillin results originating from both ASVs and OTUs at varying sequence identity cutoffs to corresponding metagenomic samples. Two previously examined datasets were assessed (human oral biopsies and rat fecal samples), along with a recently-collected human feces dataset. For all datasets, Piphillin results correlated strongly with metagenomic data using the KEGG reference, with ASVs yielding significantly higher correlation values than Piphillin data from OTUs at high identity (99 and 100%) for the human oral biopsy dataset and 100% for the human feces dataset (Fig. 1b). This is likely due to the nature of ASVs corrected by DADA2, whereby modeling is used to correct sequence errors and thus preclude spurious sequences. Since these sequences reflect the real variant, remaining equivocally strict with Piphillin parameters (i.e.*,* requiring sequences to be > 99% identical to a reference genome) maintains the necessary fidelity and leads to stronger correlation results.

The primary goal of numerous microbiome experiments is to elucidate the differential abundances of features between two candidate groups. For ASVs as input, Piphillin exhibited its best true positive rate and balanced accuracy at 99% sequence identity cutoff. This identity threshold rendered more sequences available for subsequent analyses when Piphillin was performed with ASVs as input vs OTUs. Conversely: for execution with OTUs, Piphillin exhibited its best true positive rate and balanced accuracy at 96% identity cutoff. Based on the results of our analyses, we recommend that sequence data arising from the Illumina platform be processed with DADA2 (ASVs) prior to Piphillin analysis, and that a 99% identity cutoff for Piphillin be applied downstream in the interest of maximum accuracy. For sequence reads generated using non-Illumina technologies, we still recommend processing with the OTU approach in preparation for Piphillin, with an identity threshold set to 96% for Piphillin analysis.

We then compared Piphillin performance to that of PICRUSt2, a contemporary competing bioinformatics tool. Piphillin predicts metagenomic content via direct nearest-neighbor matching between 16S rRNA gene amplicons and genomes from reference databases (i.e., KEGG or BioCyc), whereas PICRUSt2 uses hidden state prediction to infer genomic content based on genome position in a reference phylogenetic tree. To compare the accuracy of Piphillin at a 99% identity threshold to that of PICRUSt2, we executed both pipelines in parallel with DADA2-corrected ASVs from human oral biopsy samples against KEGG references. Differential abundance testing was then carried out with DESeq2, and results were compared to those arising from corresponding metagenomics techniques. While the correlation between predicted metagenomes and actual metagenomic data was not significantly different for the two different analyses of the human feces or human oral biopsy datasets, PICRUSt2 did correlate more strongly with actual metagenomics results for rat feces samples. This suggested that Piphillin and PICRUSt2 predict metagenomic functional content to an equivalent extent in many microbial samples. When comparing performance in differential abundance testing, Piphillin and PICRUSt2 exhibited comparable false positive rates, and a greater true positive rate, which in turn led to 19% greater balanced accuracy. This trend is consistent with our previous report [4]. When comparing the fold changes of individual features, both Piphillin- and PICRUSt2-predicted abundance exhibited greater fold changes than their corresponding metagenomics results, with PICRUSt2-predicted abundance change being the more extreme. This is likely due to the fact that KEGG references predict a larger number of counts of a given gene, based on taxonomy. Ultimately, Piphillin enables a user to identify more true positive features than PICRUSt2 while still minimizing false positives.

A noteworthy limitation of Piphillin is that it requires the leveraging of a robust database containing a large number of the reads relevant to a given sample, which hinders its usefulness for lesser studied samples and environments. However, as databases such as KEGG and BioCyc continue to expand, the extent of this limitation will lessen. Compared to the original KEGG database, the most recent Piphillin reference database using OTUs yielded up to 10.64% more matched reads at the recommended 96% identity threshold (Human oral biopsy; Fig. 4b). We also investigated the impact of updated references to Piphillin using ASVs with two different databases (Fig. 4a). As was observed for the OTUs, the newer KEGG database enabled many more sequences to be maintained for subsequent analysis. Similar improvements were noted in the updated BioCyc reference. As databases continue to expand, Piphillin’s prowess in predicting metagenomic functional content will continue to evolve.

Ultimately, Piphillin executed against the KEGG database yielded much greater correlation values for host-associated microbial samples than environmental samples, such as hypersaline microbial mats. This trend is consistent with our previous results. This suggests that despite remarkable advances in molecular environmental microbiology, the highly complex and reticulated nature of environmental samples is still not represented enough in reference databases. This renders them currently unable to provide the resolution required to characterize the diversity of these microbiomes. Nevertheless, Piphillin executed against the updated BioCyc database on environmental samples yielded greater correlation values to corresponding metagenomic data (Fig. 4b). This is likely due to BioCyc’s inherent emphasis placed on small-molecule metabolism from all domains of life, rather than organismal functions and human disease, as is the case with KEGG [14, 15].

Piphillin continues to be the simplest method for predicting metagenomic functional content, requiring only the input of an abundance table in csv format and a fasta file containing representative sequences or ASVs. Piphillin’s core infrastructure facilitates frequent database updates, thereby fostering continuous improvement in the utility’s resolution and robustness as new genomes are considered. Piphillin does not rely on multiple sequence alignment or phylogenetic trees, a nuance now being advocated for in other proposed tools [16]. In response to a number of requests from users, the latest release of Piphillin (i.e.*,* v7.0) outputs a tabular presentation depicting the contribution of each genome, in addition to feature and pathway abundance summary tables. Future releases will continue to integrate feedback from users, continue to update current reference databases, and may also include the addition of other reference databases. The latest release of Piphillin is available to the public [17].

## Conclusions

Here, we demonstrate Piphillin’s compatibility with 16S rRNA gene sequence data processed by both amplicon sequence variant error correction and clustering sequences into operational taxonomic units and provide detailed Piphillin parameters for data processed via each of these techniques. We also show that differential abundance testing of Piphillin-predicted metagenomic abundance data results in a larger number of true positives compared to PICRUSt2 while still minimizing false negatives. Piphillin is publicly available and provides a flexible means of leveraging 16S rRNA sequence data to predict microbial functional breadth.

## Methods

### Human oral biopsy and rat feces data

The human oral biopsy and rat feces samples were extracted and sequenced from our previous publication [4].

### Human feces data: subjects

The study subjects included in the analysis are described in [18]. Ethical approval was granted by the Cork clinical research ethics committee (APC-022 and APC-046).

### Human feces dataset: DNA extraction

An aliquot of approximately 0.2 g of feces was transferred into a tube with one 3.5 mm glass bead, 0.1 mL of 1.0 mm zirconia/silica beads and 0.1 mL of 0.1 mm glass beads (Biospec, Bartlesville, OK, USA). QIAamp Fast DNA stool kit (Qiagen GmbH, Hilden, Germany) was used for DNA extraction. First, 1 mL of InhibitEX buffer was added to the fecal samples, which were disrupted by bead-beating in a Mini-Beadbeater-24 (Biospec) for 30 s three times at maximum speed (3450 strokes/min). Samples were then heated at 95 °C for 5 mins and following this were processed according to the kit manufacturer’s instructions, resulting in genomic DNA eluted in 200 μL of ATE buffer.

### Human feces dataset: 16S rRNA PCR and sequencing

Library preparation for 16S rRNA gene amplicon sequencing was performed following the Illumina (San Diego, CA, USA) recommendations with some modifications. Briefly, aliquots of 15 ng of extracted DNA were subjected to PCR amplification of the V3-V4 hypervariable region of the 16S rRNA gene in a total volume of 30 μL. The primers used (final concentration 0.2 μM) were selected from [19] and contained the Illumina sequencing adapters (overhang nucleotide sequences) added to the gene-specific sequences (forward TCGTCGGCAGCGTCAGATGTGTATAAGAGACAGCCTACGGGNGGCWGCAG; reverse GTCTCGTGGGCTCGGAGATGTGTATAAGAGACAGGACTACHVGGGTATCTAATCC) (Eurofins Genomics, Ebersberg, Germany). PCR amplification was performed with the Phusion High-Fidelity DNA polymerase (Thermo Scientific, Wilmington, DE, USA) in a 2720 Thermal Cycler (Applied Biosystems, Foster City, CA,USA) under the following conditions: 98 °C for 30 s, followed by 25 cycles of 98 °C for 10 s, 55 °C for 15 s, 72 °C for 20 s and a final cycle of 72 °C for 5 min. The presence of the amplified 16S rRNA gene band was verified in agarose gels. PCR products were purified using Agencourt AMPure XP magnetic beads (Beckman-Coulter, Brea, CA, USA) and eluted in 52.5 μL of EB Buffer (Qiagen). After purification, 5 μL of DNA was amplified in a second PCR employing Nextera XT Index primers (Illumina). This PCR was run at 98 °C for 30 s, followed by 8 cycles of 98 °C for 10 s, 55 °C for 15 s, 72 °C for 20 s and a final cycle of 72 °C for 5 min. A second purification step with Agencourt AMPure XP magnetic beads was carried out after the Nextera PCR. The 16S V3-V4 rRNA gene amplicons containing the Nextera indexes were finally eluted in 27.5 μL of EB Buffer, and DNA concentrations were measured using the dsDNA high sensitivity assay and Qubit 3.0 fluorimeter (Thermo Scientific). Libraries were created by pooling 40 ng of each sample. Finally, the libraries were sent for sequencing at the Teagasc NGS facility (Moorepark, Cork, Ireland) on an Illumina MiSeq, utilizing a MiSeq v3 reagent kit for 2x300bp paired-end reads.

### Human feces dataset: shotgun metagenomics sequencing

Metagenomic shotgun library preparation and sequencing steps were performed using methods developed and validated by GATC Biotech (Konstanz, Germany). Briefly, after shipment in dry ice, genomic DNA integrity and quantity for quality purposes were determined by agarose gel electrophoresis, microfluidic capillary electrophoresis in a 2100 Bioanalyzer system (Agilent Technologies, Santa Clara, CA, USA) and fluorimeter measurement (Qubit). Library preparation steps included: DNA fragmentation, adapter ligation, amplification and size selection. Sequencing was performed on an Illumina HiSeq4000 in 2 × 150 bp mode.

### Reference databases

Gene copy numbers were retrieved from the Kyoto Encyclopedia of Genes and Genomes (commercial version of KEGG [20];) release 84.1 (December 2017) to create a gene feature table. From each genome sequence, 16S rRNA gene IDs were extracted using keyword “K01977” (16S ribosomal RNA) in xxx_genes.txt files (xxx represents the genome id). Corresponding fasta format 16S rRNA sequences were retrieved and filtered using a min-length of 1400 bp and a max-length of 1600 bp. The number of 16S rRNA sequences passing the length filter in each genome was recorded.

Gene copy numbers were retrieved from the commercial version of BioCyc 21.5 to create a gene feature table. From each genome sequence, 16S rRNA gene IDs were extracted using keyword “16S ribosomal RNA” as a “COMMON-NAME” in rnas.dat file in each BioCyc PGDB. Corresponding fasta format 16S rRNA sequences were retrieved and filtered using length cutoff of > 1400 bp and < 1600 bp. The number of 16S rRNA gene sequences passing the length filter in each genome was used to normalize 16S rRNA copy numbers. Gene copy numbers for each genome were retrieved, summarized by BioCyc reactions (RXNs), and formatted using a custom script for the database.

### 16S rRNA sequence processing for Piphillin

To pre-process 16S rRNA gene libraries for Piphillin functional inference, sequences were processed via one or more 16S sequence analysis methods: amplicon sequence variant error correction with DADA2 (ASVs) and/or 97% de novo OTU clustering with UPARSE (OTUs) [10, 11]. The human feces, human oral biopsy and rat feces datasets were analyzed with both methods, while hypersaline microbial mats dataset was only analyzed using OTUs as only fasta formatted files without quality scores were available for analysis.

Default settings were used to correct sequence errors into ASVs with DADA2. Trim parameters were as follows: human feces (trim left 10 bases and truncate at base 290 for both forward and reverse reads), human oral biopsy (forward read: trim left 10 bases and truncate at base 240, reverse read: trim left 10 bases and truncate at base 200) and rat feces (forward read: trim left 10 bases and truncate at base 240, reverse read: trim left 10 bases and truncate at base 225). For OTU analysis, sequences were binned into OTUs at 97% identity, a representative sequence from each OTU was selected, and the count of sequences in each OTU from each sample was tallied as previously described [21].

### Shotgun metagenomics analyses

Reads were first processed with Trimmomatic [22] to trim adapter sequences and low-quality ends (<Q20). Reads shorter than 35 bp after trimming were discarded. Contaminant sequences, like PhiX174 and sequencing primers, were removed with Bowtie2 [23]. rRNA sequences from all three domains of life were identified and removed with SortMeRNA 2.0 [24]. Host sequences were removed with Kraken [25], which used exact alignments of raw shotgun sequences to k-mers derived from the human reference genome. Filtered and translated DNA sequences were mapped separately against reference databases of all proteins within the KEGG (version 84.1) and BioCyc (version 21.5) databases using Diamond [26], where hits spanning > = 20 amino acids with > = 80% similarity were collected. In cases where one read matched these criteria against multiple proteins, only the protein or proteins (in the event of a tie) with the maximum bit score were collected.

### Inference of metagenomics by Piphillin

Piphillin was developed to utilize the most up-to-date genome databases to infer metagenomics content from 16S rRNA sequenced samples. The web version of Piphillin [17] currently supports different releases of KEGG and BioCyc. Specific details about methods in which Piphillin predicts metagenomic content is described in [4], with the only change being an update to use USEARCH version 10.0.240. Briefly, sequences (ASVs or OTUs) are searched against a database using USEARCH at a user-specified sequence identity cutoff. The genome that is the closest match to a particular 16S rRNA sequence above the identity cutoff is considered as the inferred genome for that ASV/OTU. The resulting genome abundance table is then normalized by 16S rRNA copy number of each genome before genome content is summarized at KO or RXN level for each sample.

### PICRUSt2 analysis

Unprocessed 16S rRNA gene fastq sequence data were fed to QIIME2 (Amazon EC2 image AMI 2018.2, [27]) and PICRUSt2 pipeline ([28], commit 16f29b9) to obtain functional count tables [29–32]. After import, reads were processed through QIIME2 using qiime dada2 denoise-paired, with the same trim parameters as used in DADA2 analysis described above. The ASV abundance table and representative sequences were then fed through PICRUSt2 using the KEGG database with the commands place_seqs.py, hsp.py, and metagenome_pipeline.py with default settings to create the predicted metagenomics table.

### Statistical analysis

Statistical analyses were performed in the R environment [33]. For correlation of predicted metagenomics to shotgun metagenomics, any missing values (e.g. KOs found in shotgun metagenomics but not in Piphillin-predicted results) were considered a 0 abundance before spearman correlation calculations. The DESeq2 package was used to detect differentially abundant KEGG orthologs (KO) in cancer and healthy paired human oral biopsy samples from datasets resulting from shotgun metagenomics, Piphillin, and PICRUSt2, as described previously [4].

## Data Availability

Paired cancer and anatomically matched contralateral clinically normal human oral biopsy samples are described in Schmidt et al. [16S rRNA accession number, EMBL PRJEB4953; metagenomics accession numbers, SRR3586059—SRR3586070] [34]. Hypersaline microbial mat data is described in [35, 36] [16S rRNA accession numbers, JN427016–JN539989; metagenomics accession numbers, ABPP00000000—ABPY00000000]. Human feces data is available is deposited under accession PRJNA398187 (16S rRNA accession number: SAMN11885751, shotgun accession number SAMN11885869).

## Abbreviations

ASV: Amplicon sequence variant
BA: Balanced accuracy
FPR: False positive rate
KEGG: Kyoto Encyclopedia of Genes and Genomes
OTU: Operational taxonomic unit
rRNA: Ribosomal RNA
TPR: True positive rate

## References

[1] Ivanov II, Atarashi K, Manel N, Brodie EL, Shima T, Karaoz U (2009). Induction of intestinal Th17 cells by segmented filamentous bacteria. Cell..

[2] Turnbaugh PJ, Hamady M, Yatsunenko T, Cantarel BL, Ley RE, Sogin ML (2009). A core gut microbiome in obese and lean twins. Nature..

[3] Knight R, Jansson J, Field D, Fierer N, Desai N, Fuhrman JA (2012). Unlocking the potential of metagenomics through replicated experimental design. Nat Biotechnol.

[4] Iwai S, Weinmaier T, Schmidt BL, Albertson DG, Poloso NJ, Dabbagh K (2016). Piphillin: improved prediction of metagenomic content by direct inference from human microbiomes. PLoS One.

[5] Bates KA, Clare FC, O’Hanlon S, Bosch J, Brookes L, Hopkins K (2018). Amphibian chytridiomycosis outbreak dynamics are linked with host skin bacterial community structure. Nat Commun.

[6] Mise K, Fujita K, Kunito T, Senoo K, Otsuka S (2018). Phosphorus-mineralizing communities reflect nutrient-rich characteristics in Japanese arable Andisols. Microbes Environ.

[7] Langille MGI, Zaneveld J, Caporaso JG, McDonald D, Knights D, Reyes JA (2013). Predictive functional profiling of microbial communities using 16S rRNA marker gene sequences. Nat Biotechnol.

[8] Cunningham CW (1999). Some limitations of ancestral character-state reconstruction when testing evolutionary hypotheses. Syst Biol.

[9] Jervis-Bardy J, Leong LEX, Marri S, Smith RJ, Choo JM, Smith-Vaughan HC (2015). Deriving accurate microbiota profiles from human samples with low bacterial content through post-sequencing processing of Illumina MiSeq data. Microbiome..

[10] Edgar RC (2013). UPARSE: highly accurate OTU sequences from microbial amplicon reads. Nat Methods.

[11] Callahan BJ, McMurdie PJ, Rosen MJ, Han AW, Johnson AJA, Holmes SP (2016). DADA2: high-resolution sample inference from Illumina amplicon data. Nat Methods.

[12] Callahan BJ, McMurdie PJ, Holmes SP (2017). Exact sequence variants should replace operational taxonomic units in marker-gene data analysis. ISME J.

[13] Aßhauer KP, Wemheuer B, Daniel R, Meinicke P (2015). Tax4Fun: predicting functional profiles from metagenomic 16S rRNA data. Bioinformatics..

[14] Caspi R, Altman T, Dale JM, Dreher K, Fulcher CA, Gilham F (2014). The MetaCyc database of metabolic pathways and enzymes and the BioCyc collection of pathway/genome databases. Nucleic Acids Res.

[15] Kanehisa M, Furumichi M, Tanabe M, Sato Y, Morishima K (2017). KEGG: new perspectives on genomes, pathways, diseases and drugs. Nucleic Acids Res.

[16] Edgar RC. SINAPS: Prediction of microbial traits from marker gene sequences. bioRxiv. 2017; Moran 2015:124156. doi:10.1101/124156.

[17] Piphillin server. http://piphillin.secondgenome.com/.

[18] Laserna-mendieta EJ, Clooney AG, Carretero-gomez JF, Moran C, Sheehan D, Nolan JA (2018). Determinants of reduced genetic capacity for butyrate synthesis by the gut microbiome in crohn’ s disease and ulcerative colitis. J Crohns Colitis.

[19] Klindworth A, Pruesse E, Schweer T, Peplies J, Quast C, Horn M (2013). Evaluation of general 16S ribosomal RNA gene PCR primers for classical and next-generation sequencing-based diversity studies. Nucleic Acids Res.

[20] KEGG: Kyoto Encyclopedia of Genes and Genomes. https://www.genome.jp/kegg/. Accessed 13 Dec 2017.

[21] Avilés-Jiménez F, Guitron A, Segura-López F, Méndez-Tenorio A, Iwai S, Hernández-Guerrero A (2016). Microbiota studies in the bile duct strongly suggest a role for Helicobacter pylori in extrahepatic cholangiocarcinoma. Clin Microbiol Infect.

[22] Bolger AM, Lohse M, Usadel B (2014). Trimmomatic: a flexible trimmer for Illumina sequence data. Bioinformatics..

[23] Langmead B, Salzberg SL (2013). Fast gapped-read alignment with bowtie 2. Nat Methods.

[24] Kopylova E, Noé L, Touzet H (2012). SortMeRNA: fast and accurate filtering of ribosomal RNAs in metatranscriptomic data. Bioinformatics..

[25] Wood DE, Salzberg SL (2014). Kraken: ultrafast metagenomic sequence classification using exact alignments. Genome Biol.

[26] Buchfink B, Xie C, Huson DH (2015). Fast and sensitive protein alignment using DIAMOND. Nat Methods.

[27] QIIME2. https://qiime2.org/. Accessed 26 Apr 26 2018.

[28] PICRUSt2. https://github.com/picrust/picrust2. Accessed 21 May 2018.

[29] Berger SA, Stamatakis A (2011). Aligning short reads to reference alignments and trees. Bioinformatics..

[30] Ye Y, Doak TG (2011). A parsimony approach to biological pathway reconstruction/inference for metagenomes. Handb Mol Microb Ecol I Metagenom Complem Approach.

[31] Barbera Pierre, Kozlov Alexey M, Czech Lucas, Morel Benoit, Darriba Diego, Flouri Tomáš, Stamatakis Alexandros (2018). EPA-ng: Massively Parallel Evolutionary Placement of Genetic Sequences. Systematic Biology.

[32] Louca S, Doebeli M (2018). Efficient comparative phylogenetics on large trees. Bioinformatics..

[33] R project. https://www.r-project.org/.

[34] Schmidt BL, Kuczynski J, Bhattacharya A, Huey B, Corby PM, Queiroz ELS (2014). Changes in abundance of oral microbiota associated with oral cancer. PLoS One.

[35] Kunin V, Raes J, Harris JK, Spear JR, Walker JJ, Ivanova N (2008). Millimeter-scale genetic gradients and community-level molecular convergence in a hypersaline microbial mat. Mol Syst Biol.

[36] Kirk Harris J, Gregory Caporaso J, Walker JJ, Spear JR, Gold NJ, Robertson CE (2013). Phylogenetic stratigraphy in the Guerrero Negro hypersaline microbial mat. ISME J.

