# Correction to: Piphillin predicts metagenomic composition and dynamics from DADA2- corrected 16S rDNA sequences

**DOI:** 10.1186/s12864-020-6537-9

**Published:** 2020-01-31

**Authors:** Nicole R. Narayan, Thomas Weinmaier, Emilio J. Laserna-Mendieta, Marcus J. Claesson, Fergus Shanahan, Karim Dabbagh, Shoko Iwai, Todd Z. DeSantis

**Affiliations:** 1 Informatics Department, Second Genome Inc, South San Francisco, California USA; 2 APC Microbiome Ireland, University College Cork, Co., Cork, Ireland; 3 School of Microbiology, University College Cork, Co., Cork, Ireland; 4 Department of Medicine, University College Cork, Co., Cork, Ireland

**Correction to: BMC Genomics (2020) 21:56**

**https://doi.org/10.1186/s12864-019-6427-1**

Following the publication of this article [1], the authors reported errors in Figs. 1, 2 and 5. Due to a typesetting error the asterisks denoting significance were missing from the published figures.

**Fig. 1:**
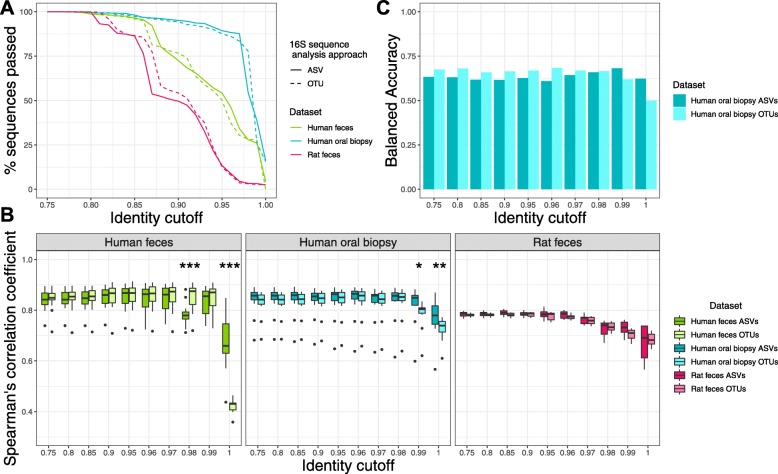
Piphillin results comparing 16S rRNA sequence analysis approaches using the KEGG database. **a** 16S rRNA gene amplicon sequences passing the identity threshold to the reference genomes. Percentage of amplicon sequences from two datasets using two different 16S rRNA sequence analysis approaches passing identity cutoffs from 75 to 100% against 16S rRNA gene sequences in the KEGG genome database. **b** Spearman’s correlation coefficient between Piphillin results and shotgun metagenomics at ten different identity cutoffs tested in Piphillin. Spearman’s correlation coefficient was calculated for each sample and mean, 1st and 3rd quartiles are depicted by the boxes. Whiskers extend to the furthest points within 150% of the interquartile range. **c** Balanced accuracy in identifying differentially abundant KOs from Piphillin against corresponding metagenomics at each identity cutoff. * indicates *p* < 0.05, ** indicates *p* < 0.001, *** indicates *p* < 0.0001

**Fig. 2:**
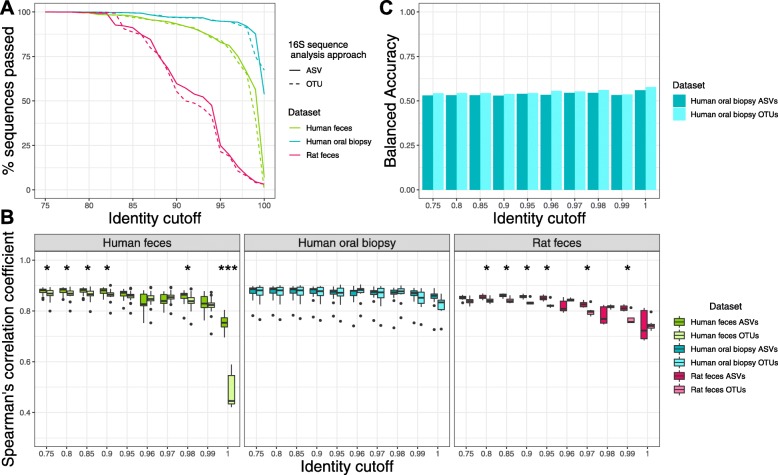
Piphillin results comparing 16S rRNA sequence analysis approaches using the BioCyc database. **a** 16S rRNA gene amplicon sequences passing the identity threshold to the reference genomes. Percentage of amplicon sequences from two datasets using two different 16S rRNA sequence analysis approaches passing identity cutoffs from 75 to 100% against 16S rRNA gene sequences in the BioCyc genome database. **b** Spearman’s correlation coefficient between Piphillin results and shotgun metagenomics at ten different identity cutoffs tested in Piphillin. Spearman’s correlation coefficient was calculated for each sample and mean, 1st and 3rd quartiles are depicted by the boxes. Whiskers extend to the furthest points within 150% of the interquartile range. **c** Balanced accuracy in identifying differentially abundant features from Piphillin against corresponding metagenomics at each identity cutoff. * indicates *p* < 0.05, ** indicates *p* < 0.001, *** indicates *p* < 0.0001

**Fig. 5:**
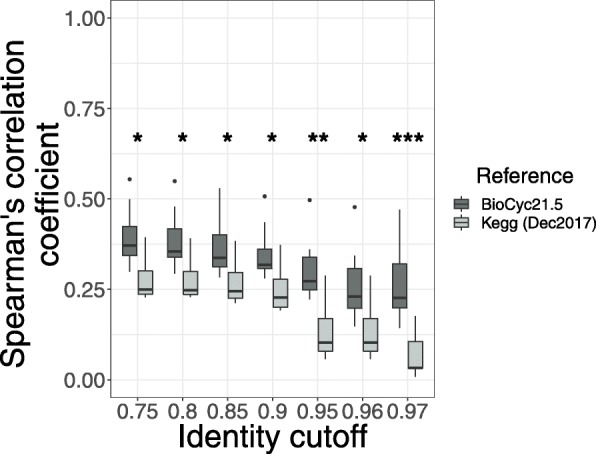
Piphillin executed with BioCyc vs KEGG reference on environmental samples. Spearman’s correlation coefficient against corresponding shotgun metagenomics results were compared the hypersaline microbial mat dataset using either KEGG and BioCyc references. Spearman’s correlation coefficient was calculated for each sample and ranges are depicted as box and whisker plots as described in Fig. 1. * indicates *p* < 0.05, ** indicates *p* < 0.001, *** indicates *p* < 0.0001

The correct figures are reproduced in this Correction article, and the original article has been corrected.
